# Supplementary material for: Illness narratives and chronic patients’ sustainable employability: The impact of positive work stories
Source: PLoS One. 2020 Feb 10;15(2):e0228581. doi: 10.1371/journal.pone.0228581 (PMC7010250; doi:10.1371/journal.pone.0228581)
Supplement: S1 File — (PDF) [file pone.0228581.s001.pdf]

Illness narratives stimuli

Condition 1: Positive work story

Original Text (Dutch)

Zon in April

Voorzichtige zonnestralen prikken door een dik wolkendek. Ze schijnen door het raam op mijn bureau, op het computerscherm. Ik kijk op de klok: 12 uur, bijna lunchtijd. Ik sta op, pak een banaan van de fruitschaal en loop naar buiten, het bos in. De bomen wuiven zachtjes op de warme lentebries, hun eerste blaadjes dansend in de wind. De grond is bezaaid met witte bloemen en het gekwetter van vogels vult de lucht. De natuur bruist van het leven. Ze lijkt wel net zo energiek als ik. Ineens besef ik welke maand het is. *April. Vijf jaar geleden…Wat was alles toen anders.* In een flits zie ik ze weer haarscherp voor me. Beelden van het ziekenhuis, van eindeloze uren in bed, van dokters, medicijnen, pijn… Huiverend wandel ik verder over het bospad, mijn gedachten bij het verleden.

Wanneer het precies begon weet ik niet meer. De buikpijn, de vermoeidheid, de diarree… Ze waren er al jaren - misschien altijd al. Het was alleen steeds wat erger geworden, als een sluipend monster dat beetje bij beetje mijn leven was gaan overnemen. Het zette zijn klauwen in feestjes, vakanties, mijn studie… Niet zeuren, dacht ik dan. Doorzetten. Iedereen heeft toch wel eens last van zijn buik? Ik ging een paar keer naar de dokter, maar die dacht dat het door stress kwam. Dat zal wel kloppen, dacht ik. *Ik was altijd zo bang om me aan te stellen.* Hoofdschuddend neem ik een hap van de banaan... Maar toen was er opeens die ene dag in april, vijf jaar geleden. De dag waarop het monster toesloeg en ik niet meer overeind kon komen door de buikpijn. Toen ging het ineens snel. Spoedonderzoek… Ontstoken darmen… Prednison. En de genadeklap, de diagnose. Een chronische darmziekte… Ik zie de dokter nog voor me. *Chronisch*, zei hij en ik wist: dit gaat nooit meer over. Dit blijft. *Voor altijd…* De wereld stortte in. Mijn studie en toekomstige carrière, vrienden, een gezin later, reizen, hobby's – niets was meer vanzelfsprekend. Ik zag mezelf al wonen in een bijstandsflat, gevangen in een doelloos, eenzaam leven. En onder al dat getob was daar steeds de buikpijn – de donkere grondtoon die alles grauw kleurde. De lente zette door en het weer werd steeds mooier, maar dat jaar klopte de lente niet.

'Hallo!' Eerst zie ik de labrador, dan het stel wat erbij hoort: de oude buren van mijn ouders. Ik aai de hond die tegen me opspringt.
'Bink! ZIT!' brult een mannenstem. De grijze vrouw loopt me lachend tegemoet. 'Dát is lang geleden! Hoe gaat het met jou?' Ze kijkt me onderzoekend aan en zegt spontaan: 'Je ziet er goed uit, zeg!'
Ik grijns. 'Het gaat ook goed. Heel goed, eigenlijk!'
'Wat fijn!' Ze kijkt me stralend aan. 'Dat is je zo gegund, na al die…na die tijd. Het is alweer jaren geleden, maar ik weet het nog goed. Wat was je bleek! Je kon amper op je benen staan. En kijk nu eens!' Ze knipoogt. 'En ik hoorde dat je een leuke baan hebt, fulltime zelfs!' Ik knik en grijns opnieuw. Bink sprint intussen het bos in. Ze knijpt me even kort in mijn arm. 'We moeten gaan. Veel succes met alles!' Nog even blijf ik staan. Ik eet mijn banaan op en gooi de schil in de bosjes. Het is inderdaad ongelooflijk hoe het nu gaat.

Je ziet er goed uit, zeg! De woorden galmen nog na in mijn hoofd. Inderdaad, wat gaat het goed. Wie had dat durven dromen? Beetje bij beetje werd het monster getemd. Ik bouwde mijn leven op, ging weer studeren, eerst in deeltijd en daarna zelfs voltijd. Het leren omgaan met mijn gezondheid ging met vallen en opstaan, maar het was toch een fantastische tijd. *Wat heb ik ondanks alles veel beleefd – vrienden, feestjes, reizen*. Als ik goed oplette, kon het allemaal. Na mijn studie solliciteerde ik en kreeg een leuke baan in een bedrijf… Niks bijstandsflat. Niks eenzaam! Niks doelloos! Als ik naar boven kijk, zijn de wolken bijna weggetrokken en stukken stralend blauwe lucht lachen me tegemoet. Ik loop terug.

Vijf jaar verder…Gelukkig maar! De ene dag gaat beter dan de andere, dat wel. Maar mijn baas weet hoe het zit en heeft er begrip voor. Na een paar mindere dagen, haal ik het werk ook altijd weer in. Tot nu toe heb ik nog geen deadline gemist. Ook mijn collega's begrijpen het. Ik ben er altijd open over geweest, ook al was dat soms best moeilijk. Maar ze reageerden fantastisch. En ze accepteren het als het even wat minder gaat. *Sterker nog: ze steunen me.* En ze maken er zelfs grapjes over. Zoals een half jaar geleden, toen mijn buik de hele dag gromde. Het ging maar door. Marijn, een nieuwe collega, keek me na een tijdje heel vreemd aan. Robin, een andere collega, moest er hard om lachen. 'Kijk maar uit, Marijn. Het grommende monster mag je niet!' Sindsdien vragen mijn collega's – inclusief Marijn - soms hoe het met het monster gaat. Ik moet glimlachen als ik er aan denk. 'Rustig,' kan ik dan de laatste tijd vaak zeggen. En vandaag al helemaal.

Het bos wordt dunner en voor me ligt het kantoor. Mijn baan is niet alleen ontzettend leuk en interessant, ik werk ook nog eens op een locatie midden in de natuur. Vaak wandel ik tijdens de lunch met collega's, maar vandaag wilde ik even alleen gaan. Ik voel de warme zon op mijn gezicht en voel me dankbaar. Het bos achter me, licht op door de felle zon. Hoe zal het over vijf jaar zijn? Ik geloof er weer in - in een stralende toekomst. Dan ben ik bij de deur. Vol vertrouwen stap ik de bedrijfshal binnen.

Free Translation in English

Sun in April

Careful sunrays pierce through a thick layer of clouds. They shine through the window on my desk, on the computer screen. I look at the time: 12 o'clock, almost lunch time. I get up, grab a banana from the fruit bowl and walk outside, into the forest. The trees wave gently in the warm spring breeze, their first leaves dancing in the wind. The soil is dotted with white flowers and the twittering of birds fills the sky. Nature busts of life. It seems as energetic as me. Suddenly I realize what month it is. *April. Five years ago… Everything was so different then*. In a flash I relive them. Images from the hospital, from endless hours in bed, from doctors, medication, pain. Shivering, I continue my walk on the forest path, my mind drifting into the past.

I cannot remember when it had started. The stomach aches, the fatigue, the diarrhea… They had been there for years - perhaps even all my life. Gradually, they had been getting worse, like a monster insidiously taking over my life. It put its claws in parties, holidays, my studies. Don't whine, I thought. Be strong. Doesn't everyone have stomach troubles sometimes? I went to the GP a few times, but he thought it was just stress. He's right, I thought. *I was always so afraid to be a wimp.* I shake my head and take a bite of the banana. But then suddenly there was that one day in April, five years ago. The day on which the monster struck

and left me unable to stand up due to the pain. All of a sudden everything happened quickly. Medical investigation. Inflamed intestines. Prednisone. The final blow, the diagnosis. A chronic bowel disease. I still see the specialist in front of me. *Chronic,* he said and I knew: this will never be over. This remains. *Forever…* The world collapsed. My study and future career, friends, a family in the future, traveling, hobbies – nothing was certain anymore. I envisioned myself living in a shelter, caught in an aimless, lonely life. My worrying these days was always accompanied by the throbbing stomach ache – the dark earth tone that colored everything gray. Spring arrived and the weather became more and more beautiful, but that year spring did not make any sense.

"Hello!" First, I see the labrador, then its owners: an old couple that used to live next door to my parents. I pat the dog that pops up against me.
"Bink! SIT!" roars the man's voice. The gray woman walks up to me, smiling. "It's been a long time! How are you?" She looks at me inquisitively and spontaneously says: "You look fantastic!"
I grin. "It's going well. Very well, actually!"
"How wonderful!" She looks at me radiantly. "You truly deserve that, after all that… that time. It's been years ago, but I remember it all so well. You were so pale! You could hardly stand up. And look at you now!" She blinks. "And I heard you found a good job, fulltime even!"
I nod and grin again. Bink sprints into the forest. She briefly touches my arm. "We have to go. Good luck with everything!"
I stand there for a while. I eat the rest of my banana and throw the peel into the bushes. It's indeed incredible how well I am doing these days.

You look fantastic! The words still echo in my head. Indeed, it is all going so well. Who had dared to dream? Step by step the monster was tamed. I rebuilt my life, started my studies again, first part-time then fulltime. Learning to cope with my health condition was a process of trial and error, yet still I have fond memories of that time. *In spite of everything, I had so many amazing experiences – friends, parties, traveling.* As long as I took good care of myself, I could have it all. After my studies, I applied for a nice job in a company. No living in a shelter! No loneliness! No aimless future! When I look up, the clouds have almost evaporated and patches of brilliant blue sky are smiling at me. I start walking back.

Five years down the line. Fortunately! Still, some days are better than others, but my boss knows about my situations and understands it. After a few less productive days, I usually catch up on work. So far, I have not missed a deadline. My colleagues understand it too. I have always been open about it, even though this was difficult sometimes. But they responded fantastically. And they accept the ups and downs of it. In fact, they support me, even by making jokes about it. Like a few months ago, when my stomach was growling all day. It went on and on. Marijn, a new colleague gave me strange looks after a while. Robin, another colleague, laughed hard. "Look out, Marijn. The grueling monster does not like you!" Since then, my colleagues – including Marijn – sometimes ask how 'the monster' is doing. I smile when I think about it. "Quiet," I can often say lately. And today as well.

The forest is thinning and the office is in front of me now. Not only is my job fun and interesting, I also work in a location in the middle of nature. I often walk with colleagues during lunchtime, but today I wanted to go for a walk on my own. I feel the warm sun on my face and feel grateful. The forest behind me lights up through the bright sunrays. How will my

life be in five years? I believe in it again – in that beautiful future. Then I am at the door. With renewed confidence, I enter the business hall.

Condition 2: Negative Work Story

Original Text (Dutch)

Kil in April

Voorzichtige zonnestralen prikken door een dik wolkendek. Ze schijnen door het raam op mijn bureau, op het computerscherm. Ik kijk op de klok: 12 uur, bijna lunchtijd. Ik sta op, pak een banaan van de fruitschaal en loop naar buiten, het bos in. Ik rits mijn jas goed dicht. Het waait flink en de boomtoppen zwiepen heen en weer, hun blaadjes klapperend in de wind. De grond is bezaaid met takken en de wind giert door het bos. De lente zet nog steeds niet door. Ik bedenk me welke maand het is. *April. Vijf jaar geleden…De maand dat alles stilstond.* In een flits zie ik ze weer haarscherp voor me. Beelden van het ziekenhuis, van eindeloze uren in bed, van dokters, medicijnen, pijn… Huiverend wandel ik verder over het bospad, mijn gedachten bij het verleden.

Wanneer het precies begon weet ik niet meer. De buikpijn, de vermoeidheid, de diarree… Ze waren er al jaren - misschien altijd al. Het was alleen steeds wat erger geworden, als een sluipend monster dat beetje bij beetje mijn leven was gaan overnemen. Het zette zijn klauwen in feestjes, vakanties, mijn studie… Niet zeuren, dacht ik dan. Doorzetten. Iedereen heeft toch wel eens last van zijn buik? Ik ging een paar keer naar de dokter, maar die dacht dat het door stress kwam. Dat zal wel kloppen, dacht ik. *Ik was altijd zo bang om me aan te stellen.* Hoofdschuddend neem ik een hap van de banaan... Maar toen was er opeens die ene dag in april, vijf jaar geleden. De dag waarop het monster toesloeg en ik niet meer overeind kon komen door de buikpijn. Toen ging het ineens snel. Spoedonderzoek… Ontstoken darmen… Prednison. En de genadeklap, de diagnose. Een chronische darmziekte… Ik zie de dokter nog voor me. *Chronisch*, zei hij en ik wist: dit gaat nooit meer over. Dit blijft. *Voor altijd…* De wereld stortte in. Mijn studie en toekomstige carrière, vrienden, een gezin later, reizen, hobby's – niets was meer vanzelfsprekend. Ik zag mezelf al wonen in een bijstandsflat, gevangen in een doelloos, eenzaam leven. En onder al dat getob was daar steeds de buikpijn – de donkere grondtoon die alles grauw kleurde. De lente zette door en het weer werd steeds mooier, maar dat jaar klopte de lente niet.

'Hallo!' Eerst zie ik de labrador, dan het stel wat erbij hoort: de oude buren van mijn ouders. Ik aai de hond die tegen me op springt.
'Bink! ZIT!' brult een mannenstem. De grijze vrouw loopt me langzaam tegemoet. 'Dát is lang geleden!' Ze aarzelt en zegt dan: 'Ik sprak je ouders laatst en hoorde dat je nog altijd zo aan het tobben bent.'
Ik slik. 'Ach ja, het gaat nog steeds niet zo goed.'
'Wat vervelend…' ze kijkt me meelevend aan. 'Ik zou het je zo gunnen dat het eindelijk eens beter gaat, na al die…na die tijd. Het is alweer vijf jaar geleden, maar ik weet nog goed dat je de diagnose kreeg. Wat was dat heftig. En wat was je bleek. Hopelijk komt er deze zomer weer wat kleur op je wangen.' Ik knik en glimlach mechanisch. Ze kijkt me vriendelijk aan. 'Ik wens je veel gezondheid toe. En: volhouden, hè! ' Ik knik opnieuw, maar mijn ogen branden. Bink sprint intussen het bos in. Ze knijpt me even kort in mijn arm. 'We moeten gaan. Veel…sterkte met alles!' Nog even blijf ik staan. Ik heb de banaan in mijn hand tot moes geknepen. Met een zucht gooi ik hem in de bosjes.

En: volhouden, hè! De woorden galmen nog na in mijn hoofd. Alsof dat zo makkelijk is! Vijf jaar tobben, dokters, diëten, alternatieve genezers, een tijdelijke baan, daarna weer ziek

thuis… Steeds de hoop - en daarna de teleurstelling. Waarom blijf ik volhouden? En waarvóór? Ik slik, maar de brok in mijn keel verdwijnt niet. Mijn buik gromt en ik grom mee - van de pijn. Het monster heeft gewonnen... Boven mij pakken naargeestige, grijze wolken samen en de eerste spetters vallen. Ik loop terug.

Nu alweer vijf jaar verder… De ene dag gaat beter dan de andere, dat wel, maar werk vinden lukt maar niet... Met veel moeite heb ik in het jaar na de diagnose mijn opleiding afgemaakt. Daarna kreeg ik een baan met een jaarcontract. Het was leuk om te werken en gaf energie, maar het bleek al gauw fysiek te zwaar. Ik werd steeds vermoeider en mijn buik werd steeds actiever. Ik ging in deeltijd werken, maar bleef achteruit gaan. Ik was er open over naar mijn collega's toe. Ze wisten zich alleen geen houding te geven, en maakten grapjes achter mijn rug. Ineens zie ik ze weer voor me op de dag dat Marijn, een nieuwe collega, bij het bedrijf kwam werken. Mijn buik gromde de hele tijd. Het ging maar door. Marijn keek me na een tijdje vreemd aan. Toen ik terugliep van de wc, hoorde ik alle collega's lachen. Ze zagen mij niet. 'Kijk maar uit, Marijn,' lachte Robin. 'Het grommende monster mag je niet!' Ik voelde me vreselijk toen ik terugliep. Uiteindelijk ging het gewoon niet meer: ik zat volledig ziek thuis. Even blijf ik staan om op adem te komen. Ik denk niet graag terug aan die tijd. Mijn contract werd niet verlengd. Uiteraard. Te riskant, vond mijn baas.

Het bos wordt dunner en voor me ligt het flatgebouw. Bijna thuis. Ik ben uitgeput. Gelukkig is mijn woning vlakbij het bos, dan kan ik er af en toe uit. Dat breekt de dag. Ik heb nog steeds geen werk kunnen vinden dat ik aankan. Hoe zal het over vijf jaar zijn? Ik durf er soms niet meer in te geloven – in de toekomst. Het begint harder te waaien. Grote druppels vallen naar beneden, spatten op mijn wangen, op mijn jas, op mijn schoenen. Ik laat het grauwe bos achter me en ga naar binnen.

Free Translation in English

Chilly in April

Careful sunrays pierce through a thick layer of clouds. They shine through the window on my desk, on the computer screen. I look at the time: 12 o'clock, almost lunch time. I get up, grab a banana from the fruit bowl and walk outside, into the forest. I zip up my coat carefully. The strong wind makes tree tops swing back and forth, their leaves flapping in the wind. The ground is dotted with branches and the wind is pouring through the forest. Spring still has not arrived. Suddenly I realize what month it is. *April. Five years ago… The month that life stood still.* In a flash I relive them. Images from the hospital, from endless hours in bed, from doctors, medication, pain. Shivering, I continue my walk n the forest path, my mind drifting into the past.

I cannot remember when it had started. The stomach aches, the fatigue, the diarrhea… They had been there for years - perhaps even all my life. Gradually, they had been getting worse, like a monster insidiously taking over my life. It put its claws in parties, holidays, my studies. Don't whine, I thought. Be strong. Doesn't everyone have stomach troubles sometimes? I went to the GP a few times, but he thought it was just stress. He's right, I thought. *I was always so afraid to be a wimp.* I shake my head and take a bite of the banana. But then suddenly there was that one day in April, five years ago. The day on which the monster struck and left me unable to stand up due to the pain. All of a sudden everything happened quickly. Medical investigation. Inflamed intestines. Prednisone. The final blow, the diagnosis. A chronic bowel disease. I still see the specialist in front of me. *Chronic,* he said and I knew:

this will never be over. This remains. *Forever…* The world collapsed. My study and future career, friends, a family in the future, traveling, hobbies – nothing was certain anymore. I envisioned myself living in a shelter, caught in an aimless, lonely life. My worrying these days was always accompanied by the throbbing stomach ache – the dark earth tone that colored everything gray. Spring arrived and the weather became more and more beautiful, but that year spring did not make any sense.

"Hello!" First, I see the labrador, then its owners: an old couple that used to live next door to my parents. I pat the dog that pops up against me.
"Bink! SIT!" roars the man's voice. The gray woman slowly walks up to me. "It's been a long time!" She hesitates and says: "I talked to your parents the other day and heard you are still struggling with your health."
I feel a lump in my throat. "Oh yes, it's still not going so well."
"I'm so sorry to hear that…" she gives me a sympathetic look. "You deserve to feel better, after all that… that time. It's been five years ago, but I remember it all so well. The time you got your diagnosis. Difficult times. You were so pale. Hopefully, this summer there will be some more color on your cheecks."
I nod and smile mechanically. She looks at me with kindness. "I wish you good health. And: don't give up!" I nod again, but my eyes are burning. Bink sprints into the forest. She briefly touches my arm. "We have to go. I truly wish you well…. The best of luck with everything."
I stand there for a while. I have squeezed the banana in my hand to a pulp. With a sigh, I throw it in the bushes.

Don't give up! The words still echo in my head. As if that's so easy! Five years of struggle, doctors, diets, alternative healers, a temporary job, then sick at home again… Every time the hope – and every time the disappointment. Why do I keep trying? For what? I swallow, but the lump in my throat does not disappear. My belly is growling and I'm joining in – from the pain. The monster has won. Above me, gloomy gray clouds breed the first splashes of water. I'm going back.

Five years down the line. Some days are better than others, but I still cannot find a job. With much effort, I completed my education in the years after the diagnosis. After this, I found a temporary job with a one-year contract. It was good to work and it gave me energy, but physically it soon became too heavy. I became increasingly tired and my illness flared up. I switched to working part-time, but my health continued to decline. I had always been open about my health issues to my colleagues, but they did not care at all and made jokes behind my back. I can still relive the day that Marijn, a new colleague, started to work for the company. My stomach was growling all day. It went on and on. Marijn gave me strange looks after a while. When I came back from the toilet, I heard all my colleagues laugh. They did not see me. "Watch out, Marijn,' laughed one of them named Robin. 'The grueling monster does not like you!" I felt terrible walking back. In the end, I was unable to work: I was sick at home every day. For a moment, I pause to catch my breath. I don't like to think about that period. My contract was not renewed. Of course, that was understandable. Too risky, according to my boss.

The forest is thinning and my flat is in front of me now. Almost home. I'm exhausted. Fortunately, my house is near the forest, so I can easily go for a little walk sometimes. It breaks the day. I still cannot find a job I can manage. How will my life be in five years? I sometimes do not dare to believe anymore - believe in a better future. The wind swells to a

storm. Large drops of rain fall down, splashing on my cheeks, on my coat, on my shoes. I leave the gray forest behind and enter the building.
