# Supplementary material for: Illness narratives and chronic patients’ sustainable employability: The impact of positive work stories
Source: PLoS One. 2020 Feb 10;15(2):e0228581. doi: 10.1371/journal.pone.0228581 (PMC7010250; doi:10.1371/journal.pone.0228581)
Supplement: S2 File — (PDF) [file pone.0228581.s002.pdf]

Supplement 2: Question Text Possible Future Work Self

Possible Future Work Self

Most people have an image or idea of themselves in their future work or career. We call this their *Possible Future Work Self*. These *Future Work Selves* can strongly differ between people.

Some people have one clear vision of themselves in their future work, others see different possible jobs as part of their future career. And while some people think of their dream job, others see a future they want to prevent, such as a boring job or becoming unemployed.

Please describe below how you see yourself in your future work. What does your future look like in terms of work or career? Feel free to describe this in your own words! Please take your time.
