# Supplementary material for: Illness narratives and chronic patients’ sustainable employability: The impact of positive work stories
Source: PLoS One. 2020 Feb 10;15(2):e0228581. doi: 10.1371/journal.pone.0228581 (PMC7010250; doi:10.1371/journal.pone.0228581)
Supplement: S3 File — (PDF) [file pone.0228581.s003.pdf]

Supplement 3: Sustainable Employability Scale

**I expect that, until retirement age, I …**	*Definitely not*	*Probably not*	*Maybe*	*Likely*	*Definitely*
… will be physically able to work.	☐	☐	☐	☐	☐
… will be psychologically able to work	☐	☐	☐	☐	☐
… will be motivated for my work	☐	☐	☐	☐	☐
… will want to keep working	☐	☐	☐	☐	☐
… will have the right knowledge and skills to work	☐	☐	☐	☐	☐
… will be able to perform well in the work that I do	☐	☐	☐	☐	☐
